# Learning rate of students detecting and annotating pediatric wrist fractures in supervised artificial intelligence dataset preparations

**DOI:** 10.1371/journal.pone.0276503

**Published:** 2022-10-20

**Authors:** Eszter Nagy, Robert Marterer, Franko Hržić, Erich Sorantin, Sebastian Tschauner

**Affiliations:** 1 Division of Pediatric Radiology, Department of Radiology, Medical University of Graz, Graz, Austria; 2 Faculty of Engineering, University of Rijeka, Rijeka, Croatia; Hanyang University, REPUBLIC OF KOREA

## Abstract

The use of artificial intelligence (AI) in image analysis is an intensively debated topic in the radiology community these days. AI computer vision algorithms typically rely on large-scale image databases, annotated by specialists. Developing and maintaining them is time-consuming, thus, the involvement of non-experts into the workflow of annotation should be considered. We assessed the learning rate of inexperienced evaluators regarding correct labeling of pediatric wrist fractures on digital radiographs. Students with and without a medical background labeled wrist fractures with bounding boxes in 7,000 radiographs over ten days. Pediatric radiologists regularly discussed their mistakes. We found F1 scores—as a measure for detection rate—to increase substantially under specialist feedback (mean 0.61±0.19 at day 1 to 0.97±0.02 at day 10, p<0.001), but not the Intersection over Union as a parameter for labeling precision (mean 0.27±0.29 at day 1 to 0.53±0.25 at day 10, p<0.001). The times needed to correct the students decreased significantly (mean 22.7±6.3 seconds per image at day 1 to 8.9±1.2 seconds at day 10, p<0.001) and were substantially lower as annotated by the radiologists alone. In conclusion our data showed, that the involvement of undergraduated students into annotation of pediatric wrist radiographs enables a substantial time saving for specialists, therefore, it should be considered.

## Introduction

The use of artificial intelligence (AI) for image analysis is one of the leading topic in the field of radiology [1–4]. Radiological AI models usually originate from annotated image data, also known as supervised AI [5] or supervised machine learning. With few exceptions, they fall into the domain of deep learning (DL) [6, 7]. DL models commonly build upon large training image sets for robust outcomes [8, 9], often containing thousands or more of different samples, such as in case of ImageNet [10], Open Images [11], or Microsoft Common Objects in Context (COCO) [12]. Corresponding radiological datasets [13, 14] are typically magnitudes smaller, since building and maintaining comprehensive deep learning systems is still challenging [8]. For image annotation a user may decide among a palette of open-source and commercial software solutions with manual and (semi-)automatic labeling techniques [8, 15–18]. However, they require area-specific expert information and development, and their implementation is often computational- and time-intensive [8]. As the workload of radiologists have increased significantly in the last decades, mainly due to the increasing number of time consuming cross-sectional images [19], alternative solutions such as with the involvement of alternative workforce in image annotation might be reasonable. Medical students have demonstrated variable learning rates in other medical contexts like surgery skills or ultrasound [20–25]. To our best knowledge, with the involvement of students in studying or annotating radiographic examinations no study has been performed so far.

The goal of the current study was to estimate the learning rate of inexperienced evaluators in labeling pediatric wrist fractures on digital radiographs. We recruited students with and without a medical background or training to annotate fractures and, thus, to assess their utility to radiologists in creating a comprehensive supervised deep learning dataset.

## Methods

We recruited nine medical and one high-school student to the study. We arranged them into four single raters and three teams of two evaluators. None of these ten individuals had specific experience in analyzing pediatric wrist fractures. Table 1 shows the particulars of these students, including previous experience in radiology or traumatology. They were instructed to manually tag all visible fractures of any age in randomly selected non-overlapping pediatric wrist digital radiography (DR) studies. Each observer processed 1,000 images, composed of 100 pictures per workday over two weeks or 10 business days.

**Table 1:** Age, sex, study duration, experience with radiography, traumatology, radiology.

Rater	Group	Student number	Gender	Year of study	Experience in wrist X-rays	Experience in Radiology	Experience in Traumatology
1	Team	1	female	5	None	Compulsory lectures	Compulsory lectures
		2	male	5	None	Compulsory lectures	Compulsory lectures
2	Team	3	female	5	None	Compulsory lectures	Compulsory lectures & clerkship
		4	female	5	None	Compulsory lectures	Compulsory lectures
3	Team	5	female	5	None	Compulsory lectures	Compulsory lectures
		6	male	5	None	Compulsory lectures, chest X-ray, Ultrasound	Compulsory lectures
4	Student	7	female	-	None	None	None
5	Student	8	female	3	None	None	None
6	Student	9	female	4	None	Compulsory lectures	Compulsory lectures
7	Control	10	female	4	None	Compulsory lectures	None

Moreover, they were asked to annotate a list of additional image tags (laterality, image projection) and classes (text, metal, bone lesion, periosteal reaction, rotational axis, foreign bodies, and soft tissue swelling) in every image, if proper to do so. We also requested the raters to judge and note the subjective difficulty of every X-ray picture on a five-point Likert scale (1 = Very easy, 2 = Easy, 3 = Neither easy nor hard, 4 = Hard, 5 = Very hard). The cumulative 7,000 student-assessed trauma radiographs were part of a comprehensive, already published dataset on pediatric trauma wrist examinations, containing 20,327 images in total [26].

Professional reporting workstations equipped with calibrated radiological 10-bit gray-level monitors RX240, RX440, or RX650 (Eizo, Ishikawa, Japan) displayed the X-ray studies in darkened reading rooms of the Division of Pediatric Radiology, Department of Radiology, Medical University of Graz. The students used the Supervisely artificial intelligence online platform (Deep Systems LLC, Moscow, Russia) to show the anonymized examinations and to label the pathologies. This platform logged numerous annotation-related parameters like overall and net annotation times or labeling durations for the available classes and tags.

Two pediatric radiologists with seven (S.T.) and eight (R.M.) years of professional experience in childhood trauma imaging re-evaluated the student interpretations by consensually obtaining the number of true/false positive/negative fracture judgments. In cases where the reference radiologists were not able to ascertain the absence or presence of a fracture, they accepted the respective student classification as either true negative or true positive. The pediatric radiologists also recorded the time necessary to correct the erroneous annotations in the image sets. Long-term average labeling time per wrist image, including all previously-mentioned classifications and objects, was 22 seconds for radiologist 1 and 21 seconds for radiologist 2. Each day, a pediatric radiologist gave constructive feedback to six of the seven raters to enable appropriate learning progress. Rater 7 (defined as control) received no specialist response during the labeling period, but after completion of the annotation procedure.

Sensitivity (true positive rate = TPR), specificity (true negative rate = TNR), positive predictive value (PPV), negative predictive value (NPV), as well as the F_1_ score [= 2*(TPR * PPV) / (TPR + PPV)] [27], were among the main parameters of interest, calculated based on the aforementioned true/false positive/negative fracture numbers. The Intersection over Union (IoU) metric (or Jaccard Index) served as a measure of bounding box accuracy [28], compared between the reference radiologists and the student-produced annotations. The literature commonly describes an overlapping area of more than 50% as good accordance between annotations by different raters [29]. A self-written Python script computed the IoU value in every image.

We performed the statistical calculations with IBM SPSS Statistics version 21 (IBM, Armonk, New York, United States of America). The dataset was analyzed with descriptive statistics and comparisons of means, specifically t-tests and ANOVAs for group comparisons. Appropriate regression curves were fitted and selected to demonstrate learning rates or visualize progression over time. P values below 0.05 were assumed to be statistically significant.

The Ethics Committee of Medical University of Graz (IRB00002556) gave an affirmative vote for the retrospective data analyses (No. 31–108 ex 18/19), waiving the necessity to obtain informed consent.

## Results

The reference radiologists diagnosed and labeled 6,072 fractures in 4,831 of 7,000 total wrist radiographs. Numbers of fractures ranged from 1 to a maximum of 3 per picture. Students marked 5,421 fractures in 4,246 images. A sum of 1,261 fractures in 1,157 images was misjudged by the raters, splitting into 305 false positives, and 956 false negatives. 1,814 images contained cast and 322 metal implants. 3,658 left, and 3,342 right radiographs were analyzed.

### Fracture detection metrics

**Fig 1:**
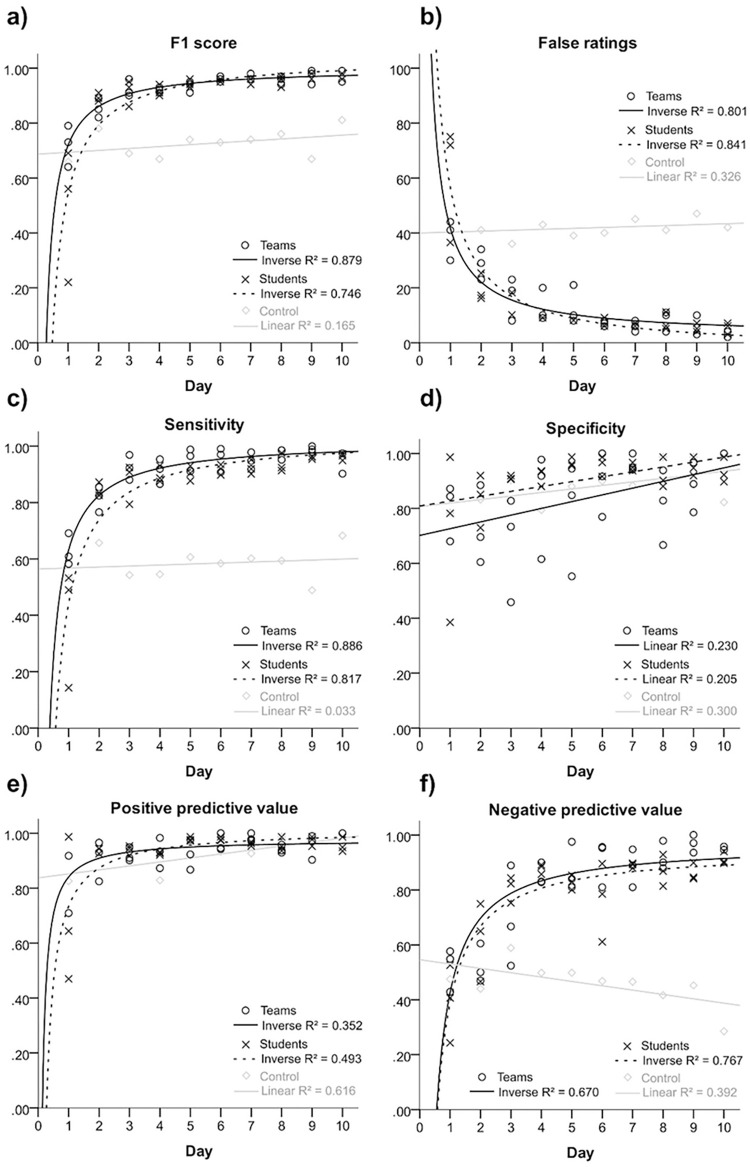
Inverse exponential curves fitted within the regression analyses of the main parameters of interest. For the control, a linear fitting was applied as baseline. R^2^ values are given for the three groups: teams (circles), individual raters (crosses), and control (boxes).

Apart from specificity (0.92 ±0.12 and 0.84 ±0.15, p = 0.022), most parameters were not significantly different between single or teams of raters in independent samples t-test: false ratings (14.20 ±17.21 and 13.90 ±11.79, p = 0.937), sensitivity (0.86 ±0.18 and 0.91 ±0.11, p = 0.248), PPV (0.94 ±0.11 and 0.94 ±0.06, p = 0.782), NPV (0.79 ±0.17 and 0.81 ±0.18, p = 0.717), and F_1_ score (0.90 ±0.15 and 0.92 ±0.08, p = 0.473). For further information refer to Fig 1A–1F and Table 2.

**Table 2:** Summary of fundamental parameters across all ten days and in total, split into the seven raters. Sums are shown for false negatives and false positives, mean values for sensitivity, specificity, and F_1_ score, and mean values plus SD for IoU.

			Day										
Parameter	Group	Rater	1	2	3	4	5	6	7	8	9	10	Total
**False negatives**	**Teams**	**1**	36	17	10	5	4	8	8	2	1	4	95
		**2**	25	16	11	5	1	1	2	1	0	2	64
		**3**	28	26	3	9	8	2	4	4	2	2	88
	**Students**	**4**	49	19	7	6	7	9	6	2	4	4	113
		**5**	60	13	7	6	8	5	5	6	4	3	117
		**6**	36	13	14	5	7	8	4	7	4	2	100
	**Control**	**7**	36	36	32	35	35	37	41	41	47	39	379
**False positives**	**Teams**	**1**	5	17	13	15	17	0	0	9	9	0	85
		**2**	5	7	8	4	7	6	2	3	3	0	45
		**3**	16	3	5	1	2	4	2	6	1	0	40
	**Students**	**4**	25	6	3	3	1	0	1	3	3	3	48
		**5**	11	3	3	3	0	1	2	0	1	0	24
		**6**	0	4	4	4	1	1	2	4	0	4	24
	**Control**	**7**	8	5	4	8	4	3	4	0	0	3	39
**Sensitivity**	**Teams**	**1**	0.61	0.82	0.92	0.95	0.97	0.91	0.92	0.99	0.99	0.90	0.90
		**2**	0.69	0.85	0.88	0.92	0.99	0.99	0.98	0.99	1.00	0.97	0.93
		**3**	0.58	0.77	0.97	0.87	0.91	0.97	0.95	0.95	0.98	0.97	0.89
	**Students**	**4**	0.49	0.83	0.91	0.89	0.93	0.90	0.90	0.98	0.95	0.95	0.86
		**5**	0.14	0.83	0.92	0.88	0.88	0.94	0.92	0.92	0.96	0.97	0.85
		**6**	0.53	0.87	0.79	0.93	0.90	0.92	0.95	0.91	0.96	0.97	0.88
	**Control**	**7**	0.53	0.66	0.54	0.55	0.61	0.58	0.60	0.59	0.49	0.68	0.59
**Specificity**	**Teams**	**1**	0.84	0.60	0.46	0.62	0.55	1.00	1.00	0.67	0.79	1.00	0.78
		**2**	0.87	0.70	0.73	0.92	0.85	0.77	0.95	0.94	0.89	1.00	0.88
		**3**	0.68	0.88	0.83	0.98	0.94	0.92	0.94	0.83	0.97	1.00	0.89
	**Students**	**4**	0.39	0.74	0.93	0.95	0.97	1.00	0.98	0.91	0.93	0.93	0.88
		**5**	0.79	0.93	0.92	0.95	1.00	0.98	0.96	1.00	0.96	1.00	0.95
		**6**	1.00	0.86	0.92	0.89	0.97	0.93	0.95	0.89	1.00	0.91	0.93
	**Control**	**7**	0.79	0.84	0.91	0.80	0.89	0.91	0.89	1.00	1.00	0.83	0.89
**F1 score**	**Teams**	**1**	0.73	0.82	0.91	0.91	0.91	0.95	0.96	0.96	0.94	0.95	0.91
		**2**	0.79	0.89	0.90	0.93	0.95	0.97	0.98	0.97	0.99	0.99	0.94
		**3**	0.64	0.85	0.96	0.92	0.94	0.96	0.96	0.94	0.98	0.99	0.92
	**Students**	**4**	0.56	0.88	0.93	0.91	0.96	0.95	0.94	0.97	0.96	0.96	0.90
		**5**	0.22	0.89	0.95	0.90	0.93	0.96	0.94	0.96	0.98	0.98	0.90
		**6**	0.69	0.91	0.86	0.94	0.94	0.95	0.96	0.93	0.98	0.96	0.92
	**Control**	**7**	0.65	0.77	0.68	0.66	0.73	0.72	0.73	0.75	0.66	0.80	0.72
**IoU metric**	**Teams**	**1**	0.33	0.34	0.38	0.39	0.37	0.59	0.45	0.40	0.43	0.40	0.40
		**2**	0.27	0.41	0.44	0.46	0.40	0.49	0.50	0.57	0.55	0.57	0.46
		**3**	0.45	0.46	0.47	0.35	0.50	0.48	0.47	0.50	0.56	0.59	0.48
	**Students**	**4**	0.22	0.41	0.38	0.51	0.62	0.54	0.46	0.67	0.50	0.51	0.47
		**5**	0.04	0.58	0.50	0.59	0.56	0.52	0.43	0.49	0.60	0.63	0.49
		**6**	0.33	0.48	0.40	0.43	0.48	0.57	0.57	0.44	0.61	0.43	0.48
	**Control**	**7**	0.19	0.34	0.33	0.21	0.37	0.28	0.34	0.43	0.27	0.40	0.31

Sensitivity (average of 0.83) decreased with higher difficulty ratings (ANOVA p<0.001). In rating 1 it was 0.83, in 2 0.90, in 3 0.82, in 4 0.79, an in difficulty rating 5 0.69. The raters perceived images with a cast more difficult, with 2.12 ±1.05 vs. 2.27 ±1.07 (p<0.001). However, the number of errors was not differing significantly (p = 0.789).

### Labeling precision

**Fig 2:**
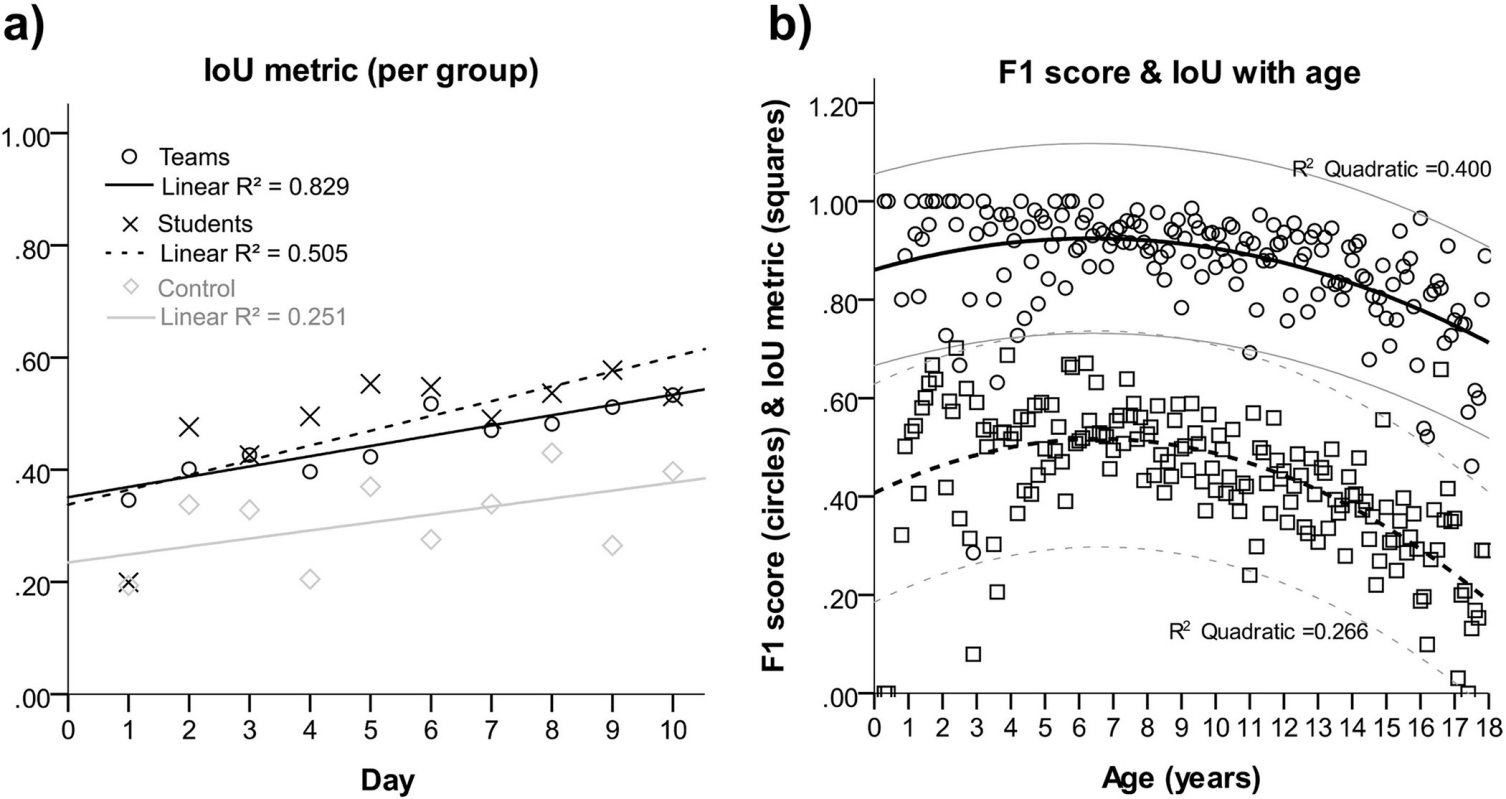
Image labeling precision during study period (a) and F1 scores and IoU metrics in relation to patient age (b). On image b quadratic curve fittings with 95% CI are displayed.

IoU increased statistically significant in all three groups over time (p<0.001), as graphically depicted in Fig 2A. IoU mean values were 0.45 ±0.28 in teams, 0.48 ±0.28 in individual raters, and 0.31 0. ±24 in control (ANOVA p<0.001). In the Bonferroni posthoc analysis, all groups were significantly different (p<0.001) with individual raters performing best.

We found that the IoU was significantly better in images with a present cast (0.49 ±0.35 vs. 0.42 ±0.38, p<0.001). We noted a similar behavior with present metal implants; IoU 0.45 ±0.29 vs. 0.41 ±0.25, p<0.001. There was no statistical significant IoU difference between left and right sides (p = 0.412), and projection (p = 0.441). The IoU was lower in images with a higher difficulty rating (ANOVA p<0.001): 0.48 ±0.28 in rating 1, 0.49 ±0.27 in rating 2, 0.41 ±0.27, 0.35 ±0.29, and 0.21 ±0.28 in difficulty rating 5.

The regression analysis revealed, that IoU and F1 score was similarly influence by patient age (Fig 2B). Image analysis was more challenging in the very young and in the older ages of life. However, the relation between F1 score and patient age was stronger (R^2^ = 0.400) than in case of IoU (R^2^ = 0.266).

### Annotation and correction times

**Fig 3:**
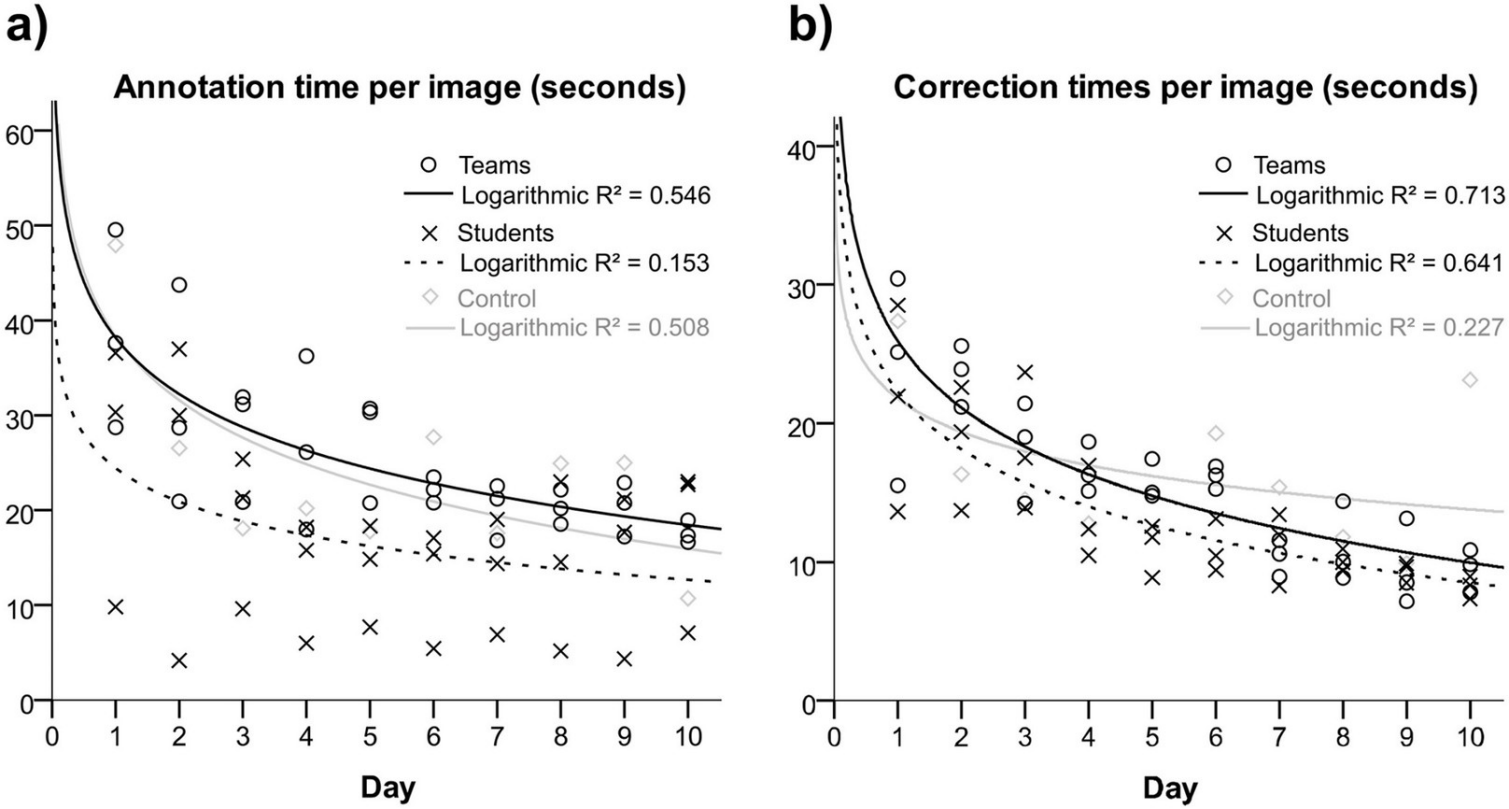
Regression analyses of annotation (a) and correction times (b). Logarithmic curve fittings are given for all three rater groups.

Times required to annotate the images decreased over the study period, as shown in Fig 3A. Mean net annotation time was 21.8 ±9.7 seconds per image; 25.2 ±8.1 seconds in teams, 17.4 ±9.5 seconds in individual raters, and 24.6 ±9.9 seconds in control. Correction time was 16.8 ±5.1 per image on average; 14.8 ±5.6 seconds for teams, 15.5 ±5.7 seconds for individual raters, 13.4 ±5.3 for control.

The number of errors in the different quarters (25 of 100 images) of the daily image sets did not differ significantly (ANOVA p = 0.218). The means and standard deviations (SD) were 1.16 ±0.40 in quarter 1, 1.11 ±0.31 in quarter 2, 1.11 ±.34 in quarter 3, and 1.15 ±.37 in quarter 4.

## Discussion

The current manuscript assessed the learning rates of students compared to board-certified pediatric radiologists in detecting and annotating childhood wrist fractures in the context of a supervised machine learning dataset generation.

The literature features only a few related studies on the learning rates of students in medical topics [21–23]. In the context of radiology, we found a few studies on ultrasound tasks [20, 24] and emergency neuroimaging [25]. Our literature inquiry did not find any comparable study on students performing image annotations on radiographics with detecting pediatric fractures in the context of supervised AI workflows.

**Fig 4:**
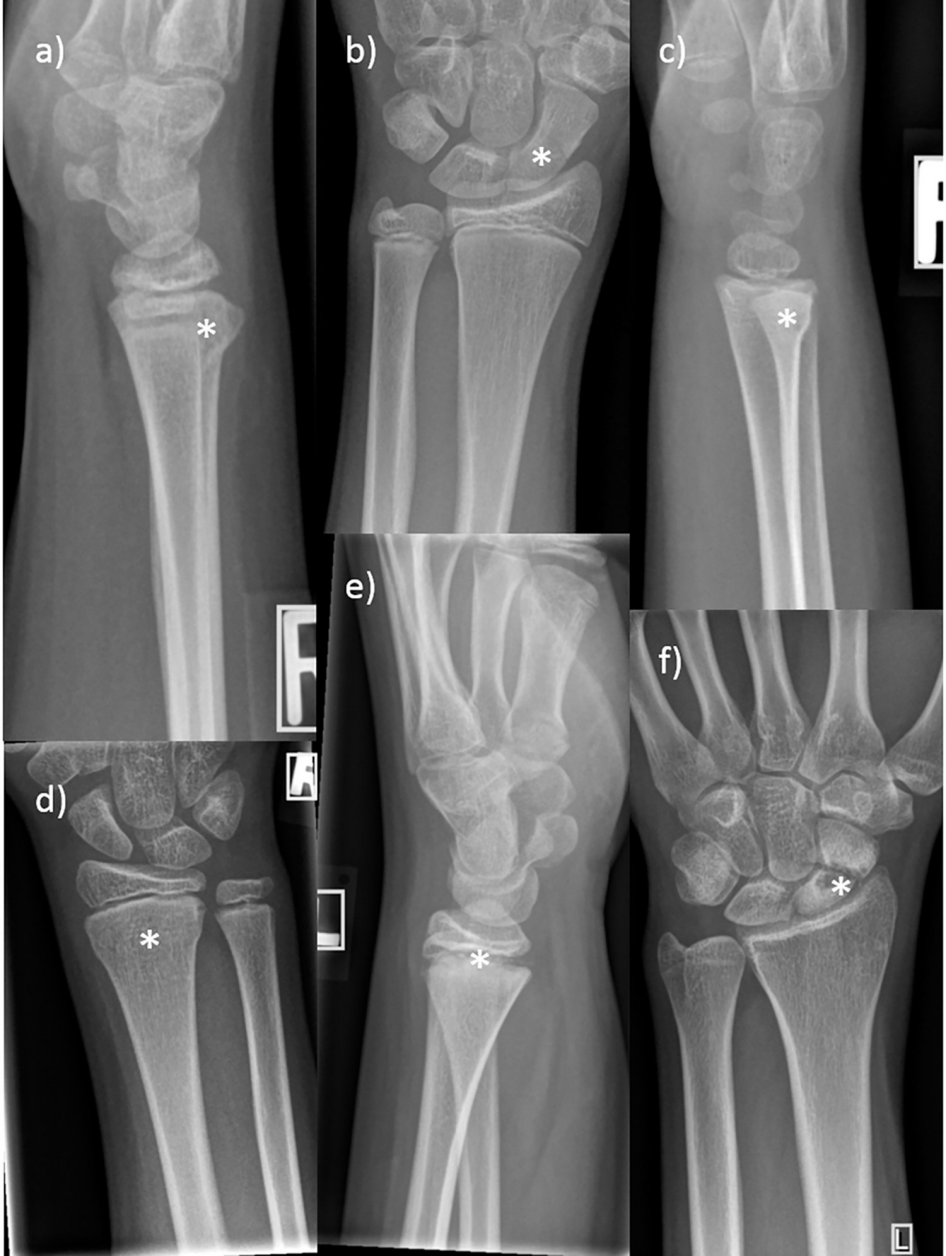
Examples of missed fractures. The stars mark the areas of bone injury. a, c & d) Dorsal compression fractures of the distal radius. b & f) Overlooked scaphoid fractures. e) Missed epiphysiolysis, Salter-Harris type 2.

We saw marked learning progress of the raters receiving professional radiologist feedback. Some of the teams and individual raters were able to exceed an F1 score of 99, while no one of them dropped below 95 on the last day. However, nobody attained an F1 score of 100 during the annotations. In contrast to the control who did not get repetitive feed-back during the annotation process, all others achieved significantly higher scores beginning from the second annotation day. Fig 4 gives examples of fractures often missed by the raters. Teams and individual raters did not exhibit relevant differences in learning rate and error patterns. Therefore, we assume that radiologists should prefer single non-expert annotators over teams with respect to responsible management of human resources. As we expected, the control demonstrated near-steady results over the study timeframe of ten days. Difficulty differences between the datasets could be the cause of the perceptible daily variance. The radiologists also gave feedback to that person after the data acquisition to capitalize on the mistakes made. In the repetitive feedback sessions, the reference radiologists systematically assessed all images of the prior rating together with the raters. The reasons for the mistakes made were debated, when possible.

Annotations times were dependent on the individual rater and demonstrated a substantial variance, as demonstrated in Fig 3A. Overall there was a decrease in annotation time per image, approaching the typical annotation durations of the radiologists of 21 seconds in a single image. A comparable established system is in common use worldwide, when consultants sign the reports of their radiologists in training. In that setting, overall student and radiologist’s annotation times together increased to about 30 seconds per picture. As compensation, the non-experts benefited by receiving feedback to achieve learning success. More importantly, the correction times for the experts decreased steadily (Fig 3B), which led to a correction time per image of about 10 seconds at day ten. This reduction means considerable time savings for the experts and could approximately double the respective annotation throughput as major bottleneck.

The study results imply that it was easy for students to learn recognition of fractures, whereas grasping the whole extension of many bone injuries was not possible for any of the raters within the study duration. While F1 scores (surrogate parameter for fracture recognition) were increasing substantially, we only saw a small increase in IoU (labeling precision) over the days. This discrepancy implies that the recognition of smaller details in the images was more challenging, e.g. even when recognized correctly, the students could not reproduce the actual extent of the seen fractures in many cases. The results of this study regarding learning performance in fracture detection may not be directly transferred to other body regions or other specific tasks. Further studies in this area appear to be legit.

**Fig 5:**
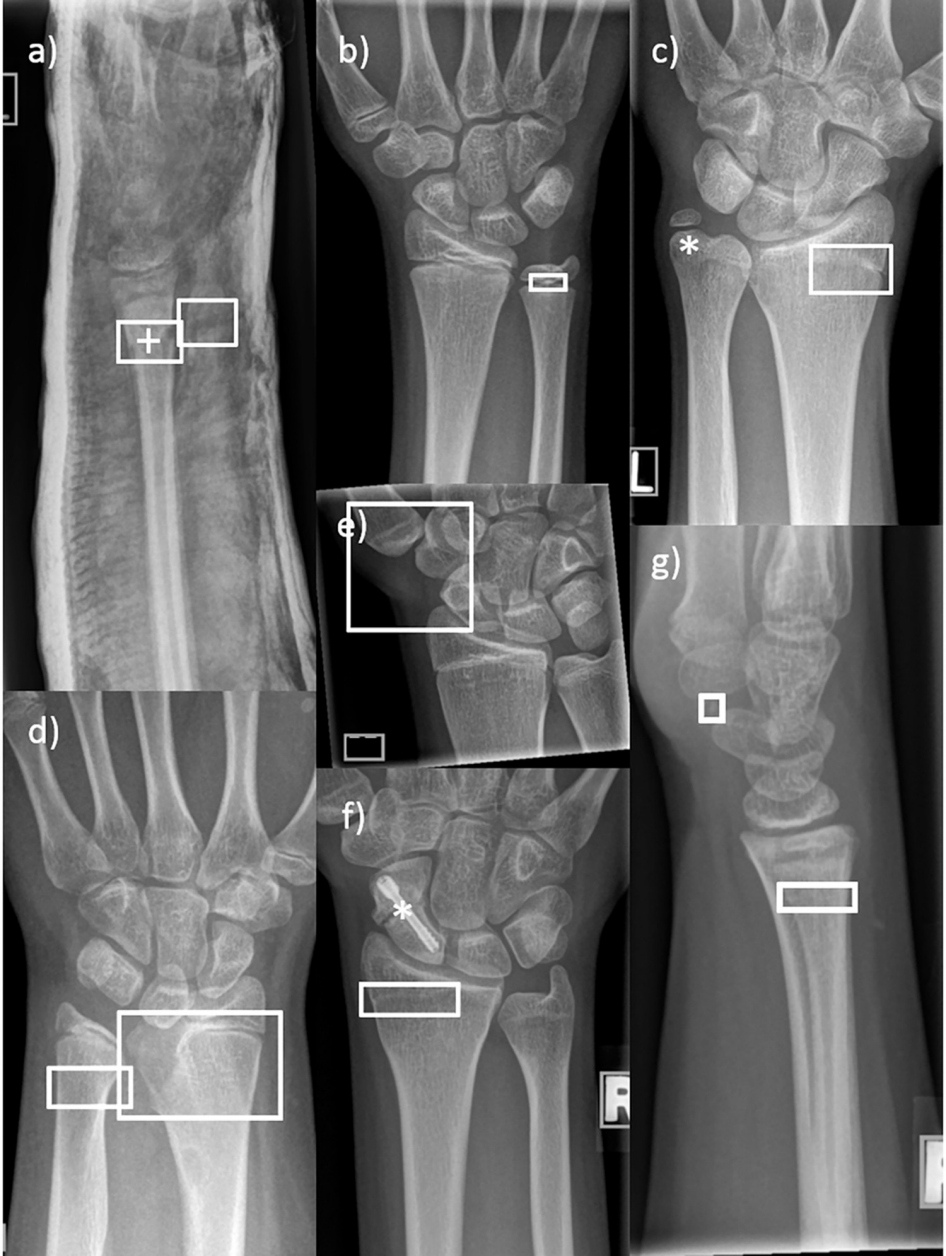
Different cases of erroneously annotated fractures in pediatric wrist radiographs. a) The cast was mistaken for a fracture. The + sign indicates the second, correctly labeled bone injury. b, c & f) Students marked the ulnar and radial growth plates as a fracture. d) A Madelung’s deformity was mimicking a fracture. e) The carpal bones were mistaken for an injury. g) A so-called Harris line thought to be a fracture. * The stars indicate missed injuries.

Surprisingly, patient age clearly influenced the number of errors and the scorings, as depicted in Fig 2B. The F1 score and the IoU decayed in teenagers and newborns, with a plateau between approximately one and ten years. Our experience indicates that fusing growth plates of the distal radius and ulna at that age (compare Fig 5) hinder the correct annotations to a certain degree. In addition, subtle fractures of the ulnar styloid process and the carpal bones were diagnosed and missed more commonly in teenagers.

Several authors proposed deep-learning algorithms to enhance the speed of image annotation by professionals as one significant bottleneck [8, 30–32]. We hypothesized, that depending on the complexity and difficulty of the labeling task, the help of inexperienced annotators accelerates the marking process. Other methods available like training a neural network on a small subsample and then applying it onto the rest [33]. This approach is known as “Human-in-the-loop” (HITL) method, which is known in many fields of artificial intelligence, also in the field of computer vision [34–36]. HITL is an alternative to the approach in this manuscript using non-specialists to relieve workload from experts when creating supervised DL record sets. It is yet undecided, which of the mentioned techniques is superior to the others.

Some limitations need to be reported and discussed. The observers faced randomly chosen datasets without overlapping examinations. That implies a certain amount of variability in difficulty to solve them correctly. A specific study set might have been more straightforward. Daily rates of true and false ratings may be affected in both directions by an "easier" or "harder" selection of studies in combination with a "lucky" or "unlucky" rater. To minimize the resulting selection bias, we decided to present the students a substantial number of 100 images per day. Also, the reference radiologists’ conditions on a particular day may influence the fracture assessment. We tried to overcome that type interference by accepting an index rating as correct if both reference radiologists were uncertain about a diagnosis. A reader should also keep the well-known fact of a reduced fracture detection sensitivity in plain radiographs in mind, which is methodically inherent. Another drawback is that we did not assess other parameters than fractures in greater detail, like bounding boxes containing text and metal, as there was a low rate of error and insignificant relevance for the project goals. Transcription errors during the correction phases are thinkable and may have occurred occasionally. However, the influence should be diminishingly small in our comprehensive dataset.

## Conclusion

In conclusion, students can help detect and label pediatric fractures around the wrist, assisting radiologists in building a supervised artificial intelligence dataset. While the error rate in fracture recognition decreased quickly under feedback, bounding box precision was not improving as much. However, after a few days of instructing, substantial time savings for the specialists are possible. Our data showed no relevant benefit for employing teams over individual non-expert raters in that setting.

## Supporting information

S1 Data (XLSX)

## Abbreviations and acronyms

AI: Artificial intelligence
DL: Deep learning
DR: Digital radiography
HITL: “Human-in-the-loop”
IoU: Intersection over Union
PACS: Picture archiving and communication system
SD: Standard deviation
